# Editorial: Early Life Epigenetic Programming of Health and Disease through DOHaD Perspective

**DOI:** 10.3389/fcell.2023.1139283

**Published:** 2023-02-10

**Authors:** Luis A. Justulin, Elena Zambrano, Thomas P. Ong, Susan E. Ozanne

**Affiliations:** ^1^ Department of Structural and Functional Biology, Institute of Biosciences, Sao Paulo State University (UNESP), Botucatu, Sao Paulo, Brazil; ^2^ Departamento de Biología de la Reproducción, Instituto Nacional de Ciencias Médicas y Nutrición, Mexico City, Mexico; ^3^ Food Research Center (FoRC), Department of Food Science and Nutrition, School of Pharmaceutical Sciences, University of São Paulo, São Paulo, Brazil; ^4^ Wellcome-MRC Institute of Metabolic Science-Metabolic Research Laboratories and MRC Metabolic Diseases Unit, University of Cambridge, Cambridge, United Kingdom

**Keywords:** DOHAD, developmental plasticity, epigenetics, adult disease, aging

## Introduction

Over the past decades, the Developmental Origins of Health and Disease (DOHaD) has been consolidated as a concept asserting the causal effects of early life exposure to environmental stressors (including malnutrition, pollutants, and stress) and the global increase in non-communicable chronic diseases observed in modern society (Gluckman et al., 2010). Although multiple mechanisms have been proposed to underlie developmental programming, epigenetic processes (including DNA methylation, histone post-translational modifications, and dysregulated non-coding RNA expression) have been described as a key mechanistic framework contributing to the non-genomic heritable increase in risk disease (Treviño et al., 2020). The articles published in this Research addressed several aspects of how early life exposure to different adverse conditions may influence health and diseases throughout the life span.

Sinzato et al. demonstrated, the negative impact of diabetes combined with lifelong high-fat diet consumption on reproductive parameters in dams, while Garcia-Santillan et al., Chavira-Suárez et al., and Simino et al. explored, respectively, the role of maternal consumption of obesogenic diet on the placental expression of nutrient transporters, methylation status in umbilical cords, and miRNA expression profile in offspring liver regeneration after partial hepatectomy. The influence of parental high-fat high-sugar diet intake on epigenetic markers and the reproductive health of male offspring was described by Sertorio et al. and Córdoba-Sosa et al. Maternal exposure to protein malnutrition was associated with the dysregulation of cell proliferation, differentiation, and impairment of epididymis development and growth (Cavariani et al.), heart fibrosis, and cardiomyocyte hypertrophy in male offspring (Folguieri et al.). An increased risk of chemically-induced mammary carcinogenesis was also reported in female offspring exposed to a maternal low protein diet (Zapaterini et al.). Wang et al., demonstrated that maternal exposure to fear maternal promoted dysregulation of the placental gene expression profile, which can contribute to placental damage and affects offspring health. Gauvrit et al. in an elegant review highlighted the association of early life exposure to stress and the development of Alzheimer’s disease (AD), emphasizing the key role of epigenetic markers on the early life origins of AD.

## Perspectives

Overall, the data published in this Research Topic presents new insights into the long-lasting effects of early life exposure to environmental stressors on offspring health. The promising results highlight the role of epigenetic markers as a key mechanistic framework underlying the Developmental Origins of Health and Disease and justify trials for early-life interventions to improve expectancy and quality of life.
